# Supplementary material for: A novel Fiji/ImageJ plugin for the rapid analysis of blebbing cells
Source: PLoS One. 2022 Apr 29;17(4):e0267740. doi: 10.1371/journal.pone.0267740 (PMC9053814; doi:10.1371/journal.pone.0267740)
Supplement: S1 Table — Table describing the measurements made by Analyze_Blebs. (PDF) [file pone.0267740.s005.pdf]

Measure	Label in Output	Label in Set Measures	Formula	Description
Aspect Ratio	AR	Shape Descriptors	$\frac{Feret_{max}}{Feret_{min}}$	The aspect ratio describes how elongated the shape of an object is. The thinner and longer the object, the higher the aspect ratio.
Circularity	Circ	Shape Descriptors	$\frac{4\pi * Area}{(Perimeter)^2}$	Circularity describes how closely an object resembles a circle by comparing it to the area equation for a circle. It does not account for any local irregularities in shape.
Solidity	Solidity	Shape Descriptors	$\frac{Area}{Convex\ Area}$	Solidity describes how closely an object resembles a circle by considering its concavity. It does account for local shape irregularities.
Roundness	Round	Shape Descriptors	$\frac{Feret_{min}}{Feret_{max}}$	Roundness describes how elongated the shape of an object is. It is the inverse of the aspect ratio, in that a longer and thinner object has a lower circularity.
Centroid	X and Y	Centroid	n/a	The centroid measure determines the center of an object based purely on the shape. It is the average of the x and y location for each pixel within the object.
Center of Mass	XM and YM	Center of Mass	n/a	Center of mass calculates the center of an object by accounting for the intensity of light within that object. It is the average of the x and y location of each pixel in the object weighted by its light intensity value.
Feret's Diameter	Feret	Feret's Diameter	n/a	Feret's diameter is the longest line that can be drawn while remaining within an object. It can also be considered the length of the longest side of a bounding rectangle for an object.
Minimum Feret's Diameter	MinFeret	Feret's Diameter	n/a	Minimum Feret's diameter is the longest line perpendicular to the Feret's Diameter that remains within the object.
Normalized Bleb Area	NormBleb-Area	n/a	$\frac{Bleb\ Area}{Cell\ Body\ Area}$	Normalized bleb area through the plugin is calculated in reference to the cell body. This measure indicates the relative proportion of blebbing area compared to non-blebbing area.
Normalized Cell Body Area	NormCell-BodyArea	n/a	$\frac{Cell\ Body\ Area}{Whole\ Cell\ Area}$	Normalized cell body area through the plugin is calculated as a proportion of the total cell area.
Normalized Largest Bleb Area	NormLarge-stBlebArea	n/a	$\frac{Leader\ Bleb\ Area}{Cell\ Body\ Area}$	Normalized bleb area through the plugin is calculated in reference to the cell body to capture the relative proportion of leader bleb area to non-blebbing area.

Distance Travelled	Distance	n/a	$\sqrt{(x_n - x_{n-1})^2 + (y_n - y_{n-1})^2}$	The plugin calculates distance travelled between two time points by using the Pythagorean theorem. The x and y values used for calculation here are derived from the centroid measure for the cell body, to exclude the contribution of bleb formation to movement. Calculations are conducted relative to the previous point, excluding the first frame from analysis.
Speed	InstSpeed	n/a	$\frac{Distance}{Time\ Between\ Frames}$	Instantaneous speed is calculated for each frame of the time lapse, tracking the distance travelled via the center of the cell body as above. The time between frames is provided by the user at start-up.
